# Supplementary material for: More than Three Decades of Bm86: What We Know and Where to Go
Source: Pathogens. 2023 Aug 22;12(9):1071. doi: 10.3390/pathogens12091071 (PMC10537462; doi:10.3390/pathogens12091071)
Supplement: Supplementary file 1 [file pathogens-12-01071-s001.zip › pathogens-2502489-supplementary.docx]

**Table S1: Research and technology development milestones.** A timeline of the most important developments regarding general technologies developed and the research milestones achieved in tick control.

| **Year** | **Technology Milestone(s)** | **Research Milestone(s)** |
| --- | --- | --- |
| 1828 | First chemical synthesis of an organic compound, i.e. urea, by Friedrich Wöhler. [200] |  |
| 1879 | First live-attenuated vaccine developed in a laboratory using *Pasturella multocida*, causative agent of chicken cholera. [16] |  |
| 1893 |  | First use of arsenic dip by cattle farmers. [201] |
| 1926 |  | First fractionation of proteins via ultracentrifugation according to molecular weight done on haemoglobin. [202] |
| 1936 |  | Arsenic resistant Rhipicephalus microplus was reported for the first time in Australia. [203] |
| 1939 |  | Demonstration of acquired immunity to ticks: Guinea pigs vaccinated with larval extracts were protected against *Dermencator Varabilis* ticks. [36] |
| 1946 |  | Introduction of organochlorines for tick control (e.g. DDT). [204] |
| 1955 |  | Introduction of organophosphates into the market for tick control (e.g. dioxathion). [205] |
| 1956 |  | First report of resistance to organochlorides. [205] |
| 1975 | -First study regarded as proteomics. [206]  -Original DNA array: colony hybridization method. [207] | Registration of the formamidine amitraz for use in the control of ticks. [208] |
| 1977 | -DNA sanger sequencing invented. [209]  -The first functional recombinant protein (somatostatin) was produced in *E. coli.* [210] | Introduction of synthetic pyrethroids for use in tick control. [211] |
| 1979 | Smallpox finally eradicated through intense global vaccination program initiated by the WHO. [212] | -First vaccination of cattle with extracts from *Dermacentor andersoni* ticks. [40]  -First report of resistance to the pyrethroid permethrin in *R. microplus* from Australia. [211] |
| 1981 | First effective vaccines against parasitic diseases in livestock: DICTOL and DIFIL. Irradiated larval vaccines against *D. viviparous* infection in cattle and *D. filaria* in sheep. [213] | Introduction of macrocyclic lactones into the acaricidal market. [214] |
| 1982 | The commercialization of the first protein produced through recombinant DNA technology: human insulin produced in *E. coli* (Genetech Inc.). [215] |  |
| 1983 | Development of polymerase chain reaction (PCR) invented. [216] |  |
| 1985 | Phage display discovered, when George Smith showed that peptides can be expressed on the surface of filamentous bacteriophage. In 1988, Stephen Parmley and George Smith described biopanning for affinity selection and demonstrated that recursive rounds of selection could enrich for clones present at 1 in a billion or less. In 1990, Jamie Scott and George Smith described creation of large random peptide libraries displayed on filamentous phage. [137] |  |
| 1986 | First commercialized vaccine using yeast (*Saccharomyces cerevisiae*) as a production system: Hepatitis B vaccine by Merck Sharp & Dohme Research Laboratories. [17] | First vaccination of cattle against *R. microplus* using extracts derived from adult females. [43] |
| 1987 |  | -Introduction of fipronil into the acaricidal market. [217, 218]  -First report of resistance to organophosphates in *R. decoloratus* and *Amblyomma variegatum* ticks from Zambia. [219] |
| 1989 |  | -Identification of Bm86 as a protective antigen from *R. microplus.* [45]  -Cloning and expression of Bm86 in *E. coli.* [19] |
| 1993 |  | -Registration of the Bm86 vaccine Gavac^®^ (Cuba). [59]  -Bm86 localization on the surface of tick midgut cells. [50] |
| 1994 |  | -Commercialization of the first Bm86-based tick vaccine TickGARD^®^ produced in *E. coli* (Australia). [54]  -Introduction of fluazuron into the acaricidal market. [220] |
| 1995 | Invention of expression library immunization (ELI): protection against mycoplasma infection. [116] | -Commercialization of TickGARD^®^ Plus where Bm86 was produced in *Pichia pastoris.* [47]  -First report of amitraz resistance in *R. microplus* ticks from Brazil. [221] |
| 1996 | DNA microarrays invented. [117] | The first Bm86-based combination vaccine with Bm91: enhanced efficacy demonstrated. [62] |
| 1997 |  | Large-scale production of the recombinant Bm86 vaccine Gavac^®^ in *Pichia pastoris.* [59] |
| 1998 | RNA interference discovered: *Caenorhabditis elegans.* [118] | First study evaluating cross-species protection of Bm86-based vaccines (Gavac^®^ ) against *R. annulatus.* [94] |
| 1999 |  | Evaluation of Gavac^®^ and TickGARD^®^ in field trials: first observed variation in efficacy. [60, 68] |
| 2000 | Launch of the first next generation sequencing technologies (Lynx Therapeutics: Massively parallel signature sequencing). [119] | Identification of Bm95 and commercialization of Gavac Plus^®^ (Cuba). [70] |
| 2001 |  | First report of resistance to the macrocyclic lactone avermectin in *R. microplus* ticks from Brazil. [222] |
| 2002 |  | -First application of RNA interference in ticks. [130]  -First epitope mapping of Bm86 and the first Bm86-based peptide vaccines. [84] |
| 2003 |  | Identification of protective antigens for control of *Ixodes scapularis* using ELI: identification of Subolesin. [131] |
| 2005 | Development of the nanopore sequencing technology and founding of the Oxford Nanopore Technologies Ltd. [223] | The *I. scapularis* genome project. [120] |
| 2006 | First studies to report RNA sequencing. [126, 127] | The *R. microplus* genome project. [121] |
| 2007 |  | -First gene silencing of *R. microplus* protective antigens: Bm86, Bm91 and Subolesin. [133]  -First application of RNAseq in ticks (vitellogin mRNA *Dermacentor variabilis*). [127]  -First report of fipronil resistant *R. microplus* ticks from Uruguay. [224] |
| 2008 | First reports of RNAseq used to map and quantify transcriptomes. [225-227] | First application of microarrays in ticks: *R. microplus.* [128] |
| 2009 |  | -Efficacy of Bm86 orthologs Ba86 [77] and rHaa86 [102] demonstrated in homologous vaccine trials.  -Bm86 found to be expressed at different levels in different tick species. [109] |
| 2010 |  | -Discontinuation of TickGARD^®^ and TickGARD^®^ Plus*.* [47]  -Identification of Bm86 homologue -ATAQ. [110] |
| 2013 |  | The first reported transcriptomes for ticks (*I. scapularis [* [228]] and *R. microplus* [229, 230]. |
| 2014 | Roll out of the first hand-held nanopore sequencing devices. [223] | The first report of fluazuron resistant *R. microplus* ticks from Brazil. [231] |
| 2016 |  | Assembled genome for *I. scapularis*. [232] |
| 2017 |  | Draft genome assembly for *R. microplus*. [122] |
| 2018 |  | Commercialization of Go-Tick^®^ or Tick-Vac^®^ (Columbia) tick extract vaccine and Bm86-based vaccine Bovimune-ixovac^®^ (Mexico). [106] |
| 2021 | Malaria vaccine R21: the first to immunize against parasitic infection. [233] | Assembled genomes for *R. microplus* and *R. annulatus.* [123] |
| 2023 |  | High-quality *I. scapularis* genome. [124] |
